# CD56 positive central nervous system plasmacytosis in a patient with refractory CD56 negative primary plasma cell leukaemia

**DOI:** 10.1002/jha2.271

**Published:** 2021-08-03

**Authors:** Jun Yong, Jeremy Schofield, David Lawton, Gillian Brearton

**Affiliations:** ^1^ Department of Haematology Clatterbridge Cancer Centre 65 Pembroke Place Liverpool Merseyside L7 8YA UK; ^2^ Department of Haematology Liverpool University Hospital NHS Foundation Trust Prescot Street Liverpool Merseyside L7 8XP UK; ^3^ Haemato‐Oncology Diagnostics Service (HODS) Liverpool University Hospital NHS Foundation Trust Prescot Street Liverpool Merseyside L7 8XP UK

**FIGURE 1:**
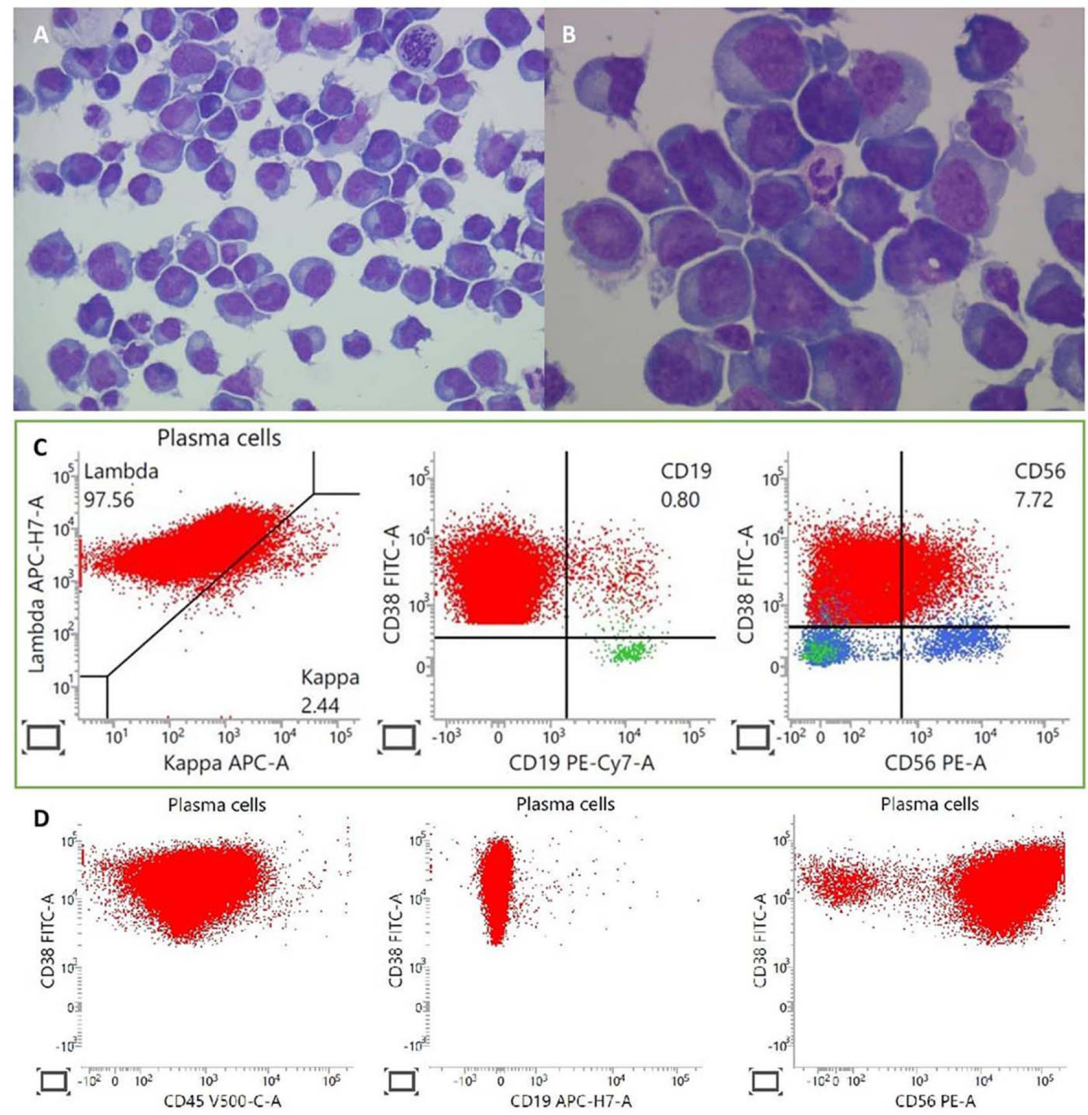


A 44‐year‐old woman with refractory plasma cell leukaemia (PCL) develops new‐onset tonic‐clonic seizures, having only recently completed four cycles of intensive combination chemotherapy with Bortezomib, Cisplatin, Cyclophosphamide, Dexamethasone, Doxorubicin, Etoposide, and Thalidomide. Urgent non‐contrast computed tomography scan of the head was unremarkable. Cerebrospinal fluid sampling revealed florid plasma cell infiltration, with occasional mitotic forms and plasmablasts seen (Figure 1A, 50x oil immersion objective lens; Figure 1B, 100x oil immersion objective lens). In contrast to known circulating CD56 negative (−) plasma cells (Figure 1C, green box), her cerebrospinal fluid plasma cells were identified to be CD56 positive (+) (Figure 1D, bottom row).

PCL is a rare and aggressive plasma cell dyscrasia with a tendency for extramedullary involvement. Central nervous system (CNS) involvement is, however, exceedingly rare. Without proven effective treatment, PCL with CNS plasmacytosis confers an abysmal prognosis. CD56, a membrane glycoprotein of the immunoglobulin superfamily, is negative in 80% PCL. Its absence discriminates PCL from multiple myeloma, where it is frequently aberrantly upregulated. Its absence has also been associated with CNS involvement. The identification of a CD56+ CNS plasmacytosis, in the context of progressive CD56‐ primary PCL, is therefore unexpected and its significance unclear. Further research is required to elucidate the role of CD56 in PCL.

All authors reviewed and finalized the contents of this pape

